# Proteome Analysis of Human Neutrophil Granulocytes From Patients With Monogenic Disease Using Data-independent Acquisition*[Fn FN2]

**DOI:** 10.1074/mcp.RA118.001141

**Published:** 2019-01-10

**Authors:** Piotr Grabowski, Sebastian Hesse, Sebastian Hollizeck, Meino Rohlfs, Uta Behrends, Roya Sherkat, Hannah Tamary, Ekrem Ünal, Raz Somech, Türkan Patıroğlu, Stefan Canzar, Jutte van der Werff Ten Bosch, Christoph Klein, Juri Rappsilber

**Affiliations:** From the ‡Bioanalytics, Institute of Biotechnology, Technische Universität Berlin, 13355 Berlin, Germany;; §Dr. von Hauner Children's Hospital, Department of Pediatrics, University Hospital, LMU Munich, 80337 Munich, Germany;; ¶Wellcome Centre for Cell Biology, University of Edinburgh, Edinburgh EH9 3BF, UK;; ‖Children's Hospital, Hematology-Oncology, Technical University Munich, 80804 Munich, Germany;; **Acquired Immunodeficiency Research Center, Isfahan University of Medical Sciences, Isfahan, Iran;; ‡‡Schneider Children's Medical Center of Israel, Petah Tikva, Sackler School of Medicine, Tel Aviv University, Israel;; §§Department of Pediatrics, Division of Pediatric Hematology & Oncology, Erciyes University, Kayseri, Turkey;; ¶¶Pediatric Department A and Immunology Service, Jeffrey Modell Foundation Center, Edmond and Lily Safra Children's Hospital, Sheba Medical Center, Tel Hashomer, Sackler Faculty of Medicine, Tel Aviv University Tel Aviv, Israel;; ‖‖Gene Center, Ludwig-Maximilians-Universität München, Munich, Germany;; ‡‡‡Department of Pediatric Hematology Oncology, UZ Brussel, Brussels, Belgium

**Keywords:** Immunology*, Omics, Diagnostic, Personalized medicine, Proteogenomics, data-independent acquisition, neutrophil granulocyte, primary immunodeficiency diseases, systems medicine, whole exome sequencing

## Abstract

Neutrophil granulocytes are critical mediators of innate immunity and tissue regeneration. Rare diseases of neutrophil granulocytes may affect their differentiation and/or functions. However, there are very few validated diagnostic tests assessing the functions of neutrophil granulocytes in these diseases. Here, we set out to probe omics analysis as a novel diagnostic platform for patients with defective differentiation and function of neutrophil granulocytes. We analyzed highly purified neutrophil granulocytes from 68 healthy individuals and 16 patients with rare monogenic diseases. Cells were isolated from fresh venous blood (purity >99%) and used to create a spectral library covering almost 8000 proteins using strong cation exchange fractionation. Patient neutrophil samples were then analyzed by data-independent acquisition proteomics, quantifying 4154 proteins in each sample. Neutrophils with mutations in the neutrophil elastase gene *ELANE* showed large proteome changes that suggest these mutations may affect maturation of neutrophil granulocytes and initiate misfolded protein response and cellular stress mechanisms. In contrast, only few proteins changed in patients with leukocyte adhesion deficiency (LAD) and chronic granulomatous disease (CGD). Strikingly, neutrophil transcriptome analysis showed no correlation with its proteome. In case of two patients with undetermined genetic causes, proteome analysis guided the targeted genetic diagnostics and uncovered the underlying genomic mutations. Data-independent acquisition proteomics may help to define novel pathomechanisms in neutrophil diseases and provide a clinically useful diagnostic dimension.

## Abstract (precis)

Data-independent acquisition proteomics was used to study proteome changes of naive human neutrophils in rare monogenic diseases affecting their functions. Neutrophils of patients with mutations in the neutrophil elastase gene *ELANE* demonstrated global proteome dysregulation, whereas chronic granulomatous disease and leukocyte adhesion deficiency had modest effects on the respective neutrophil proteomes. Proteomics then guided targeted genetic assays to resolve two clinical cases with undetermined genetic causes, highlighting the usefulness of mass spectrometry-based clinical diagnostics.

## Graphical Abstract

**Figure:**
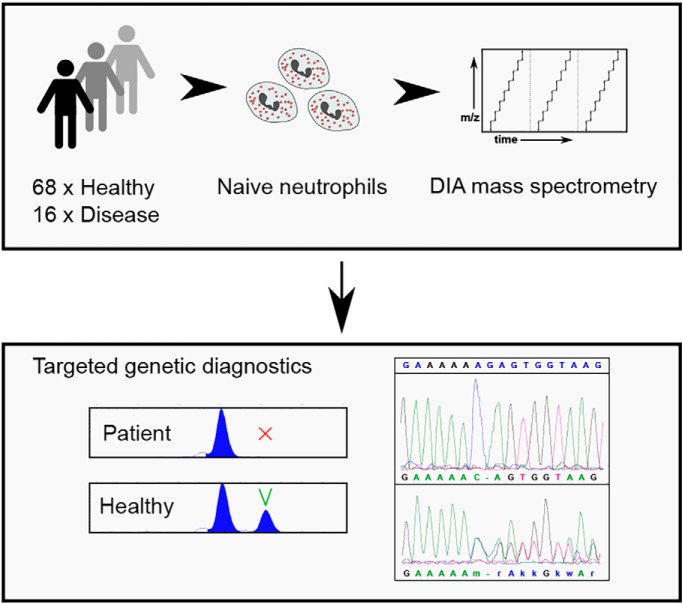

**Highlights**

- Data-independent acquisition proteomics analysis of naive neutrophils from patients with rare monogenic diseases.
- Proteomics analysis helps guide targeted genetic diagnostics of patients for which routine clinical diagnostics proved inconclusive.

Neutrophil granulocytes constitute the most abundant population of nucleated cells in human blood. Whereas their role in defense against microbes has been known for more than a century, their sophisticated roles in tissue remodeling, cancer and chronic inflammation has emerged only recently (1, 2).

Rare diseases of neutrophil granulocytes may affect their differentiation and/or function. Severe congenital neutropenia (SCN)^1^ comprises a heterogeneous group of monogenic disorders characterized by aberrant premature apoptosis of myeloid progenitor cells (3). Chronic granulomatous disease (CGD) results from mutations in one out of five subunits of the NADP-oxidase complex (4). Leukocyte adhesion deficiency (LAD) causes aberrant transendothelial migration of neutrophil granulocytes because of integrin defects (5).

In striking contrast to their prominent role in health and disease, there are very few validated diagnostic tests assessing the function of neutrophil granulocytes. Although quantitative studies (*i.e.* differential blood counts) are among the most common laboratory tests, validated qualitative studies are virtually absent, except for measurement of NADPH-oxidase activity for CGD and expression of defined cell surface markers to diagnose LAD.

Genetic sequencing assays, based on defined panels, exome, or whole genome sequencing are the gold standard for the diagnosis of monogenic diseases, yielding conclusive results in up to 25–50% of patients (6, 7). To capture the complexities of diseases and to increase the diagnostic yield, additional omics technologies and data integration may be useful. This has recently been shown for strategies associating genome and RNA-sequencing in rare neuromuscular diseases (8, 9). We hypothesized that in-depth proteome-analysis may also provide additional cues for diagnosis of monogenic diseases, such as rare defects of neutrophil granulocytes. In the past, several laboratories have published data on the proteome of healthy neutrophil granulocytes (10–23), however all but one (10) quantified significantly fewer proteins compared with our study. We here systematically analyze proteome changes in neutrophils from patients with different monogenic diseases and demonstrate the usefulness of next-generation proteomics for guiding clinical genetic diagnostics.

## EXPERIMENTAL PROCEDURES

### 

#### 

##### Experimental Design and Statistical Rationale

The total number of analyzed neutrophil samples was 84 (68 healthy controls and 16 patients). The patient samples were measured without replication. The size of the healthy control group allowed to average out biological variation. The rationale for choosing the large healthy control group was that it allowed to better estimate parameters of the Gaussian curves fitted to protein expression profiles for outlier detection in the two unclear clinical cases. Differential protein expression analysis was performed with limma R package (24), while blocking for batch effects.

##### Patient Cohort

Patients were recruited from pediatric centers in Germany (LMU University, Dr. von Hauner Children's Hospital; and TU University, Department of Pediatrics), Turkey (Erciyes University, Fevzi Mercan Children's Hospital, Kayseri), Iran (Isfahan University, Imam Hossein Children's Hospital, Isfahan) and Israel (Schneider Children's Medical Center of Israel, Tel Aviv). Informed consent was given by the parents or legal guardians in accordance with the Declaration of Helsinki and European legislation. Children were asked for their informed assent. The study was approved by the LMU Munich ethics committee as well as ethics boards of local clinical institutions.

##### Extraction and Purification of Neutrophils

Blood was drawn from patients and healthy donors into EDTA-containing collection tubes (clinical standard tubes for anticoagulation, Sarstedt, Nümbrecht, Germany, 04.1915.100) and immediately processed within a 4 h time window. Neutrophils were isolated with the MACSexpress human neutrophil isolation kit (Miltenyi, Bergisch Gladbach, Germany, 130-104-434) according to the vendor's protocol. For total erythrocyte depletion the MACSxpress Erythrocyte Depletion Kit (Miltenyi, 130-098-196) was used according to the vendor's protocol. After isolation, neutrophils were twice washed in PBS (Gibco, Paisley, Scotland, UK, 14190250), microscopically counted in a hemocytometer and divided into aliquots of 1 × 10^6^ or 2.5 × 10^5^ cells. A cytospin stained with May-Grunwald Giemsa for cell purity control was prepared when possible. After pelleting, the supernatant was removed and 5 μl of 25x protease inhibitor was added (Roche, Penzberg, Germany, 04693159001). Cells were then frozen in a −80 °C freezer before being transferred to storage in liquid nitrogen until final proteome preparation.

##### Neutrophil Sample Preparation for Mass Spectrometry

Purified neutrophil samples were processed with the Filter-Aided Sample Preparation (FASP) method as follows: ∼10^6^ cells were lysed using 50 μl of SDS lysis buffer (0.5% SDS, 0.1 m DTT in 0.1 m Tris-HCl pH 7.6) and sonicated for 30 s using a Branson Ultrasonics 250A analog sonifier (10% duty cycle, energy level 1). Samples were then heated for 5 min at 95 °C in a heating block. Subsequently, 150 μl of UA buffer (8 m urea in 0.1 m Tris-HCl pH 8.5) were added to the samples to a total volume of 200 μl, loaded onto 0.5 ml Microcon 30 kDa-cutoff Ultracel membrane filters (Merck, Germany, catalog number MRCF0R030) and spun down in an Eppendorf 5418 centrifuge for 20 min at 14,000 rcf. Next, 200 μl of UA buffer was added and the centrifugation repeated. Subsequently, 50 μl of IAA buffer (0.05 m iodoacetamide in UA buffer) were added to the filters and incubated in darkness for 20 min at room temperature. Next, two washing steps using 150 μl and 200 μl of UA buffer were performed, each time spinning down the samples for 20 min at 14,000 rcf. As a final washing step, the samples were washed twice with 200 μl of ABC buffer (50 mm ammonium bicarbonate in ddH2O) and spun down as described above.

The filters with washed samples were then transported to new collector tubes and MS-grade trypsin (Thermo Fisher, Germany, catalogue number 90057) in digestion buffer (1 m urea in 0.1 m Tris-HCl 8.5) was added in 1:100 ratio. The filters were wrapped in parafilm to prevent drying and placed in a wet chamber for overnight digestion at 37 °C.

Finally, the samples were spun down for 15 min at 14,000 rcf, 50 μl of ABC buffer were added to the filters and the centrifugation step repeated. The samples were then acidified to pH ∼2.5 using 20% TFA (trifluoroacetic acid). The peptide yield was estimated using Thermo Fisher NanoDrop 2000c.

The samples were cleaned-up and desalted using a C18-StageTip approach as described in (25) and stored at −20 °C.

##### Generation of Spectral Library: SCX Chromatography

To create a comprehensive neutrophil spectral library, selected healthy donor and patient peptide samples were eluted from StageTips using 80% acetonitrile (ACN) in 0.1% formic acid, dried in Eppendorf Concentrator plus 5305, reconstituted in SCX buffer A (5 mm K2HPO4 in 10% ACN) and pooled. 100 μg of pooled peptides were separated on a Shimadzu LC-20AD HPLC system using a PolyLC PolySULFOETHYL-A SCX column (100 × 2.1 mm, 3 μm beads, 300 Å pores) with a 12 min nonlinear gradient of SCX buffer B (1 m KCl, 5 mm K2HPO4 in 10% ACN) while collecting fractions every 15 s. The fractions were then concentrated, reconstituted in 0.1% TFA and desalted using C18-StageTips. Lower complexity fractions were pooled together prior to mass spectrometric analysis.

##### Generation of Spectral Library: Data-dependent Acquisition

The spectral library SCX fractions were analyzed on Thermo Fisher QExactive HF mass spectrometer coupled to a Dionex UltiMate 3000 HPLC system using a 50 cm C-18 Thermo Fisher EasySpray column (catalog number ES803), heated to 50 °C. Roughly 2 μg of peptides was loaded onto the column for each run. All samples contained spiked-in iRT peptides (Biognosys, Switzerland, catalog number *K_i_*-3002-2) for retention-time alignment.

A 135-min gradient was used as follows: the flow rate was set to 300 nl/min, start at 2% buffer B (80% ACN in 0.1% FA) with a linear increase to 35% B for 90 min followed by a linear increase to 41% until 102 min and to 99% B until 104 min with a hold at 99% B until 120 min for washing. The gradient was then reduced to 2% B at 120 min and held at 2% for 15 min for column re-equilibration.

A Top10 DDA method was used as follows: 1 full MS1 scan between 350 and 1300 *m*/*z* at resolution of 120,000, with AGC target of 3e6, maximum injection time (IT) of 50 ms and a default charge state of 2. Ten most intense peaks were selected for MS2 fragmentation using resolution 15,000, AGC target of 1e5 and a maximum IT of 80 ms. The isolation window was set to 1.6 *m*/*z*, fixed first mass to 100 *m*/*z* and a normalized collision energy (NCE) to 30. Additionally, a dynamic exclusion of 30 s was set.

##### Generation of Spectral Library: Raw Data Processing

Peak lists obtained from DDA MS/MS spectra were identified using X! Tandem Vengeance (2015.12.15.2) (26), Andromeda version 1.5.3.4 (27), MS Amanda version 1.0.0.7501 (28), MS-GF+ version Beta (v10282) (29) and Comet version 2016.01 rev. 3 (30). The search was conducted using SearchGUI version 3.2.19 (31).

Protein identification was conducted against a complete Uniprot SwissProt human protein sequence database (state on June 27, 2016). The decoy sequences were created by reversing the target sequences in SearchGUI. The identification settings were as follows: trypsin, specific, with a maximum of 2 missed cleavages. 10.0 ppm as MS1 and 20 ppm as MS2 tolerances; fixed modifications: carbamidomethylation of C (+57.021464 Da); variable modifications: acetylation of protein N-term (+42.010565 Da), oxidation of M (+15.994915 Da); fixed modifications during refinement procedure: carbamidomethylation of C (+57.021464 Da); variable modifications during refinement procedure: pyrolidone from E (–18.010565 Da), pyrolidone from Q (–17.026549 Da), pyrolidone from carbamidomethylated C (–17.026549 Da). Peptides and proteins were inferred from the spectrum identification results using PeptideShaker version 1.16.11 (32). Peptide Spectrum Matches (PSMs), peptides and proteins were validated at a 1% False Discovery Rate (FDR) estimated using the decoy hit distribution. All engine-specific settings were set kept as default. The list of peptides identified in this study can be found in the supplemental Table S6.

##### Data-independent Acquisition of Neutrophil Proteomes

The data-independent acquisitions were performed on the same equipment as the spectral library DDA measurements using the exact same chromatography conditions. Each patient sample was measured once because of the size of the cohort. All the samples contained spiked-in iRT peptides (Biognosys, catalogue number *K_i_*-3002–2) for retention-time alignment. The mass spectrometry settings were as follows: one MS1 scan was performed between 350 and 1300 *m*/*z* at resolution of 120,000, AGC target of 5e6 and a maximum IT of 100 ms, followed by ten 12.5 *m*/*z* MS2 windows, ten 37.5 *m*/*z* MS2 windows and a final single 450 *m*/*z* MS2 window (21 MS2 windows total). In case of all the MS2 windows the resolution was set to 30,000, AGC target to 1e6, maximum IT to “auto” and the collision energy to 30. Both MS1 and MS2 scans were recorded in profile mode. The DIA method resulted in a median of 7 data points per peak. The mass spectrometry proteomics data have been deposited to the ProteomeXchange Consortium via the PRIDE (33) partner repository with the data set identifier PXD010701.

##### Protein Identification and Quantification

Biognosys Spectronaut 11 was used for DIA search and protein quantification. The DIA raw files were converted into HTRMS format using Biognosys HTRMS converter.

Our sample-specific SCX spectral library containing 119193 precursors, 87757 peptides and 7977 proteins, was imported. Minimum of 3 up to 6 best fragments per peptide were used. The DIA search and quantification were performed with the following settings: using precision iRT and nonlinear iRT calibration, MS1 and MS2 mass tolerance strategy were set to “Dynamic,” XIC RT extraction window was set to “Dynamic.” Precursor FDR was set to 1% and protein FDR was set to 5% (using decoy method set to “inverse”). Data filtering was set to “Qvalue percentile 0.5,” cross-run normalization was set to “Qvalue complete.” Peptide quantification was performed using mean precursor quantity (using up to 3 top precursors) and the area under the MS2 signal. Protein quantification was performed using mean peptide quantity (using up to 3 top peptides per protein). Protein inference was set to automatic. All settings for our Spectronaut analyses can be found in the Spectronaut experiment file (.sne) uploaded to the PRIDE archive with raw files.

As a final filtering step, known contaminants according to MaxQuant (34) were removed from the list of quantified proteins.

##### Differential Protein Expression Analysis

Limma R package (24) was used to perform differential protein expression analysis using empirical Bayes moderation. The log2-transformed expression values were normally distributed. In order to increase the power of the analysis, the extra term (sample processing date) was added to the model for blocking as a mean of batch-effect control. The expression matrix used in this analysis was not processed by the ComBat algorithm. Only proteins identified by two or more peptides were used for differential abundance testing. Hits with Benjamini-Hochberg P-adjusted value <0.01 were considered statistically significant.

##### Gaussian Model Fitting for Protein Expression Anomaly Detection

Log2-transformed protein expression values in healthy donors were used to fit a Gaussian model for each protein using R package MASS (35). Proteins quantified with at least three peptides were used for increased stringency. Protein expression values of each of the two patients were used to calculate probability that a given protein is expressed similarly to healthy donors. Finally, proteins were sorted by increasing probability of having a healthy expression value. Proteins with assigned probability <0.01 were used for more detailed manual inspection.

##### Sanger Sequencing

Patient DNA was extracted from full blood samples with Qiagen DNeasy blood and tissue Kit (Qiagen, Hilden, Germany, catalog number 69504). PCR reaction was performed using OneTaq DNA polymerase kit (New England Biolabs, Frankfurt a.M., Germany, catalog number M0480X), all with the same cycler conditions (5′: 95°, 30”: 95°, 30”: 55°, 45”: 72°, 5′: 72°). Before sequencing samples were treated with Illustra ExoProStar (Merck, Darmstadt, Germany, catalog number US78220) for removal of primers and dNTP. Sequencing was performed by Eurofins Genomics Germany, data were analyzed using SeqMan Pro of the DNAstar software suite. The primers for *ELANE* were designed in-house, whereas the primers for *RAB27a* were kindly provided by Meeths *et al.* (36). The complete list of sequencing primers can be found in the supplemental Table S7.

##### Whole Exome Sequencing

High throughput sequencing was performed by NextSeq500 with 2 × 150 bp reads. The exomes were enriched using SureSelect Human All Exon Kit Agilent V6+UTR. BWA (37) (version 0.7.15) algorithm for short Illumina reads was used to align the short reads to the human reference genome GRCh37. The Genome Analysis Tool Kit (38) (GATK version 3.6) was used for analysis of the whole exome sequencing data. All algorithms and parameters were chosen according to the GATK best practice pipeline. Functional Annotation was performed with the Variant Effect Predictor (39) (VEP version 85). Final variants were filtered with a custom database encompassing gnomAD (40) and GME (41) following a rare homozygous and compound heterozygous inheritance. SIFT (42) and PolyPhen-2 (43) were used as provided by VEP.

##### RNA Sequencing

Neutrophils from 22 healthy donors were isolated using the same approach as for the proteomics samples. RNA was isolated with the RNAeasy plus mini-kit from Qiagen (catalog number 74134) according to the vendor protocol. Magnetic oligo-dT beads (NEB) were used for mRNA enrichment starting with 1 to 10 ng total RNA with RIN values above 9. The NEBNext Ultra II directional RNA Library prep Kit (NEB) was used to prepare RNA-seq libraries according to the manufacturer's protocol. Paired-end sequencing was performed with 2 × 75 cycles on an Illumina NextSeq 500 (Care-for-Rare Genomics Facility at the Dr. von Hauner Children's Hospital). The short reads were aligned with STAR (44) version 2.5.0a to the human reference genome GRCh37.p13 with basic two-pass method. The counts were then generated using the featureCounts program from the subread toolkit (45) version 1.5.1. The counts were normalized for sequencing depth and RNA type using DESeq2 (46). Median transcripts-per-million (TPM) values were calculated by normalizing the median gene read counts to the length of the union of all possible exons coded by a given gene. The raw RNAseq data have been deposited into Gene Expression Omnibus (47) (GEO) under accession number GSE118644.

##### Gene Ontology Term Enrichment Analysis

The GO term enrichment was performed using DAVID (48) online tool using proteins quantified in the healthy neutrophils as background. The GO BP DIRECT terms were used for analysis.

##### Data Analysis and Plotting

All data were analyzed using R 3.4 and plotted using ggplot2 R package (49). Differential protein expression analysis was performed using the limma R package (24). Single-patient protein expression anomaly detection was performed using R MASS package (54).

## RESULTS

### 

#### 

##### Proteome Analysis of High-purity Neutrophil Samples

**Fig. 1.**
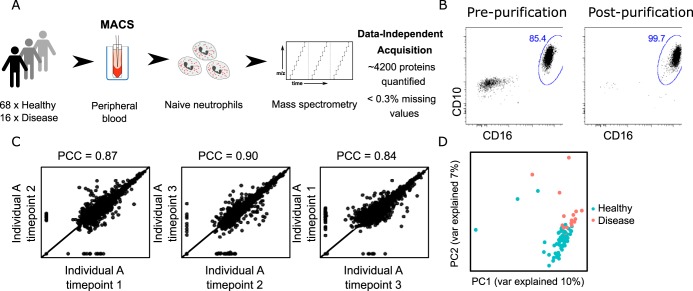
**Mass spectrometry-based workflow for neutrophil proteome profiling.** *A*, Neutrophil granulocyte samples were extracted from peripheral blood of healthy donors and patients, purified and subjected to data-independent acquisition mass spectrometry. *B*, Flow cytometry showed high neutrophil purity (>99%) after isolation. *C*, Pearson correlation analysis of samples from the same individual obtained at three different timepoints shows high level of correlation among biological replicates. PCC, Pearson correlation coefficient. *D*, Principal component analysis demonstrates good separation of healthy and diseased individuals using full protein expression profiles.

First, we set up an experimental flow allowing for consistent, robust, and reliable analysis of purified neutrophil granulocytes. Using magnetic-bead based negative selection we isolated neutrophil granulocytes with >99% purity from 16 patients with quantitative or qualitative defects (supplemental Table S1) and 68 healthy control individuals (Fig. 1*A*, 1*B*). Although 14/16 patients had a genetically confirmed molecular diagnosis of neutrophil deficiency (CGD: *n* = 5, SCN: *n* = 6, LAD: *n* = 3), in two patients (one with NADPH-oxidase deficiency, one with congenital neutropenia associated with albinism) routine genetic workup by exome sequencing did not yield a specific defect in any gene known to cause congenital neutropenia or CGD. A set of 4154 proteins was quantified across all samples using data-independent acquisition (DIA) mass spectrometry and neutrophil-specific spectral libraries generated by us. The estimated protein copy numbers per cell using the proteomic ruler approach (50) and the full list of quantified proteins in each sample are reported in the supplemental Table S2. To estimate the variability in protein expression measured by our approach, we compared proteomes of the same healthy individual sampled over three different timepoints spanning several months. As expected from DIA proteomics (51), we observed a high level of agreement among replicates with a mean Pearson correlation coefficient of ∼0.87 (Fig. 1*C*). After correcting for batch effects related to sample processing using the ComBat algorithm (52), we looked at the separation of neutrophil proteomes of healthy donors and patients using principal component analysis (PCA) (Fig. 1*D*). The groups were clearly separated along the two first principal components, explaining in total ∼17% of the total variability in the data.

##### Neutrophil mRNA Levels Are Not Well Correlated with Protein Levels

**Fig. 2.**
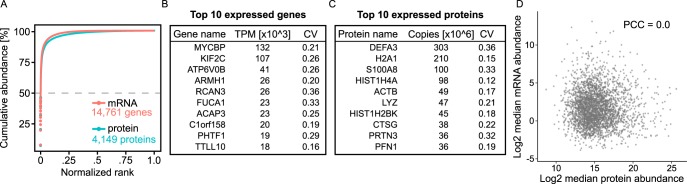
**Protein and transcript expression profiles in healthy neutrophils.** *A*, Cumulative abundances of proteins and protein-coding transcripts follow a similar pattern. Only seven proteins account for ∼50% of the total protein copy number in neutrophils. *B*, Top 10 expressed protein-coding transcripts in healthy donor neutrophils. TPM, transcripts per million; CV, coefficient of variation. *C*, Top 10 expressed proteins in healthy donor neutrophils. CV, coefficient of variation *D*, We observed no correlation (PCC = 0.00) of transcriptome and proteome in healthy naive neutrophil granulocytes. PCC, Pearson correlation coefficient.

Next, we looked at correlation of protein expression with expression levels of the respective mRNAs in the healthy naive neutrophils. We sequenced the transcriptomes of 22 healthy donor neutrophil samples and compared cumulative protein and protein-coding transcriptome abundances (Fig. 2*A*). Interestingly, only seven proteins accounted for 50% of the total protein copy number. This suggests that the neutrophil proteome is dominated by a very few highly expressed proteins, with the antimicrobial proteins DEF3A, S100A8, LYZ, and CTSG being among the most abundant. A similar dynamic range can be seen for the protein-coding transcriptome (top three abundant transcripts: MYCBP, KIF2C, ATP6V0B). However, the top expressed protein-coding transcripts did not correspond to the top expressed proteins (Fig. 2*B*, 2*C*). The healthy donor neutrophil mRNA abundances were reported in supplemental Table S3.

We found no correlation between mRNA and protein levels (Pearson correlation coefficient of 0.00; Fig. 2*D*). This result is in sharp contrast to correlation coefficients obtained in other cells, which typically range from ∼0.4 to 0.8 (53, 54) and agrees with previous observations (13, 16, 55). Naive neutrophils have been shown previously to have reduced transcriptional activity (56). It is therefore possible that the naive neutrophil transcriptome is not reflective of its function, but rather prepared to initialize gene expression programs on activation, as suggested by (56).

For this reason, we focused on in-depth analysis of proteome rather than transcriptome data.

##### Differential Protein Expression in Neutrophil Granulocytes from Patients with Monogenic Diseases

**Fig. 3.**
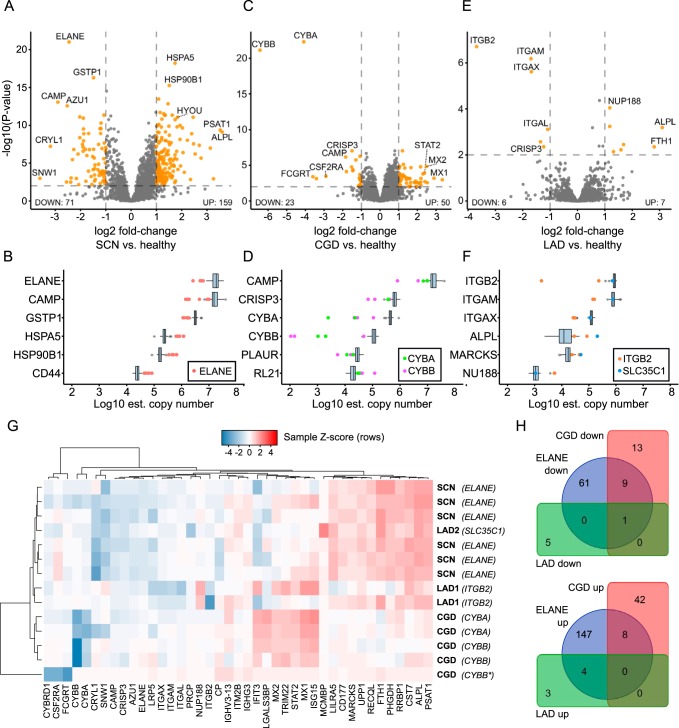
**Differential protein expression in SCN, CGD and LAD.** *A*, Volcano plot illustrating changes in neutrophil granulocyte protein expression between SCN-ELANE (*n* = 6) and healthy individuals (*n* = 68). *X*-axis showing log2 fold change compared with healthy control, *Y*-axis showing -log10(*p* value). A total of 71 proteins were significantly underexpressed in *ELANE*-mutant cells, 159 were significantly overexpressed. Points marked in orange have adjusted *p* values <0.01 and absolute fold-change >2. *B*, Top six significantly differentially expressed proteins in SCN, ordered by decreasing mean estimated log10 copy number. The differentially expressed proteins span four orders of magnitude of abundance. *C*, Differential protein analysis of CGD cases *versus* healthy individuals. The most downregulated proteins are members of the flavocytochrome b558 complex affected in CGD, namely CYBA and CYBB. Interferon-1-responsive network members (MX1, MX2, STAT2) were significantly up-regulated in CGD. Points marked in orange as in Fig. 3*A. D*, Top six significantly differentially expressed proteins in CGD, ordered by decreasing mean estimated log10 copy number. *E*, Differential protein analysis of LAD cases *versus* healthy control. Only modest proteome-level effects could be detected. The most downregulated proteins were integrins which are members of the affected protein family (ITGB2, ITGAM, ITGAX). *F*, Top six significantly differentially expressed proteins in LAD, ordered by decreasing mean estimated log10 copy number. *G*, Heatmap of protein expression levels in all analyzed cases normalized to expression in healthy individuals and z-transformed within each sample. Samples and proteins were ordered using hierarchical clustering. Dendrograms show relative Euclidean distances between samples or proteins. Samples tend to be clustered close together as expected by genetic analysis, except for the one LAD2 case. Very homogenous expression profiles can be seen for the six SCN samples and four CGD samples. CYBB*, *CYBB* splice donor variant. *H*, Venn diagram comparing differentially expressed proteins in SCN-ELANE, CGD, and LAD (note the low degree of overlap between diseases).

We asked the question to what extent the composition of the proteome differed among patients with severe congenital neutropenia because of mutations in *ELANE* and healthy individuals. As shown in Fig. 3*A*, the proteome of *ELANE*-mutated neutrophil granulocytes showed 71 significantly underexpressed and 159 overexpressed proteins. As expected, ELANE was one of the most underexpressed proteins (4.5-fold). In addition, other primary granule antimicrobial proteins, such as azurocidin (AZU1, 4.5-fold) and cAMP receptor protein (CAMP, 5-fold) were also markedly underexpressed. In the group of significantly overexpressed proteins, we identified several endoplasmic reticulum (ER) heat-shock proteins (DNAJB1, HSP90B, HSPA5, HYOU1, SDF2L1, PDIA4, MANF), indicative of proteostatic stress response. Another prominent group of significantly overexpressed proteins were ribosomal proteins (RPL9, RPL10, RPL11, RPL19, RPL21, RPL22, RPL31, RPL32, RPL38, RPS23) and mitochondrial proteins (SPTLC2, ABCB10, EIF2A. LRPPRC, CYCS, SLC25A1, LONP1, PUS1, LETM1, TOMM22, GRPEL1, HSPA9, SHMT2). Several transcription factors were differentially expressed (decreased levels: TCEAL3, ZNF22, increased levels: LRPPRC, SND1). It is currently not known whether this reflects differences in maturation stages or a specific response to the disturbed subcellular environment in *ELANE*-mutated neutrophil granulocytes. Fig. 3*B* shows the relative abundance of the top 6 proteins with the most significantly changed expression values. The most enriched Gene Ontology (GO) Biological Process (BP) terms among the up-regulated proteins were “translational initiation” (adj. *p* value: 9.8 × 10^−11^), “nuclear-transcribed mRNA catabolic process, nonsense-mediated decay” (adj. *p* value: 2.8 × 10^−5^) and “protein folding” (adj. *p* value: 8.3 × 10^−4^). The most significant BP term enriched among down-regulated proteins was “proteolysis” (adj. *p* value: 5.6 × 10^−2^), a term which encompasses the neutrophil antimicrobial proteins like ELANE and AZU1. In contrast to the proteome of *ELANE*-mutated neutrophil granulocytes, the analysis of neutrophil granulocytes from CGD patients (*n* = 5) revealed fewer striking differences when compared with healthy individuals (Fig. 3*C*). Interestingly, the two proteins with the lowest expression levels were CYBA and CYBB (down-regulated 16-fold and 84-fold, respectively). This suggests that mutations in either CYBA or CYBB are destabilizing the membrane-bound heterodimeric complex, whereas the expression of the three cytosolic members of the NADPH-oxidase complex (NCF1, NCF2, NCF4) is not affected (supplemental Fig. S1). Several secondary granule proteins, such as CAMP, LTF, and CRISP3, were also decreased in abundance, albeit to a lower degree. The most enriched GO BP terms among the upregulated proteins in CGD were: “type I interferon signaling pathway” (adj. *p* value: 4.4 × 10^−11^), “defense response to virus” (adj. *p* value: 1.3 × 10^−7^) and “complement activation, classical pathway” (adj. *p* value: 1.1 × 10^−2^). The most significant BP term enriched among downregulated proteins in CGD was: “cell redox homeostasis” (adj. *p* value: 2.1 × 10^−2^) which encompasses the affected proteins CYBA and CYBB together with CAMP and LTF. The proteomes of patients with LAD (2× *ITGB1*, 1× *GFTP*/*SLC35C1*) were minimally disturbed when compared with healthy individuals and showed ITGB2 and related members of the integrin family (ITGAM, ITGAX, ITGAL) as proteins with strongest underexpression. Moreover, the antimicrobial protein CRISP3 was found to be expressed at lower levels than controls (supplemental Fig. S2). No GO BP terms were enriched among the up-regulated proteins in LAD. The most enriched GO BP terms among the downregulated proteins in LAD were: “integrin-mediated signaling pathway” (adj. *p* value: 7.6 × 10^−4^), “leukocyte migration” (adj. *p* value: 7.1 × 10^−4^), “extracellular matrix organization” (adj. *p* value: 2.0 × 10^−3^) and “cell adhesion” (adj. *p* value: 1.8 × 10^−2^). All differentially expressed proteins have been reported in the supplemental Table S4.

##### Comparison of Protein Expression Profiles of SCN, CGD, and LAD

We compared the similarity of differentially expressed proteins in SCN-ELANE, CGD, and LAD (Fig. 3*G*). As expected, most of the patient samples clustered together according to their clinical phenotypes. There was however one exception: the one LAD2 (*SLC35C1*) sample was clustered with the SCN group owing to its different genetic background than the two LAD1 cases and protein expression changes more resembling SCN cases than LAD1.

In the heatmap one can observe multiple distinct protein clusters. One prominent cluster of proteins was the interferon 1-response pathway (STAT2, MX1, MX2, TRIM22, IFIT3), overexpressed in four CGD cases, one LAD1 case and one SCN case, but mostly unchanged in other SCN cases and the other two LAD cases. All patient neutrophils showed underexpression of granule proteins (CAMP, CRISP3, AZU1), with SCN cases exhibiting the strongest difference. Finally, a prominently underexpressed CYBA/CYBB (flavocytochrome b558) cluster of four CGD cases can be observed, with the fifth CGD case demonstrating very minimal underexpression of those proteins. Next, we directly compared all significantly over- and underexpressed proteins of the three clinical phenotypes for overlaps (Fig. 3*H*). Overall, we observe only minimal overlap of significantly dysregulated proteins between the three diseases.

##### Proteome Analysis Guiding Molecular Diagnosis

**Fig. 4.**
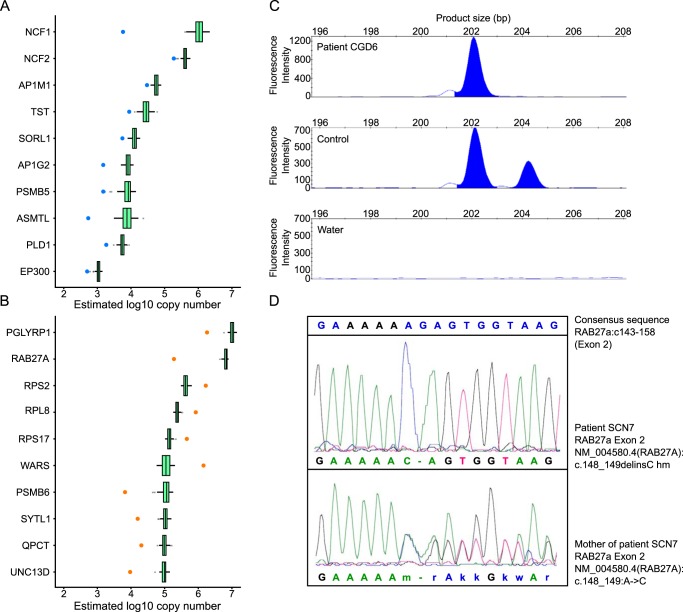
**Proteome analysis guiding molecular diagnosis.** *A*, Top 10 deregulated proteins in patient CGD6. *B*, Proteome deregulation analysis for the other neutropenia case without known genetic mutation. The protein-level analysis found a significant underexpression of RAB27A protein (by two orders of magnitude) and its interaction partner, SYTL1. *C*, Gene-scan analysis using gene- and pseudogene-specific fluorescent primers in patient CGD6. Note two normal NCF1 alleles (product size 204 bp) and 4 NCF1 pseudogene alleles (product size 202 bp) in a healthy control which result in the physiologically normal 2:1 signal ratio. In patient CGD6, only 4 pseudogene alleles but no normal NCF1 alleles are present. DNA-free water served as a negative control. *D*, Sanger sequencing of the *RAB27a* gene in patient SCN7 and his mother revealed a homozygous mutation in exon 2 (NM_004580.4(RAB27A):c.148_149delinsC) in the patient. The patient's mother was a heterozygous carrier of the mutation.

Two patients in our cohort had clinical signs and symptoms of neutrophil granulocyte deficiency (one patient with CGD confirmed by negative nitro blue tetrazolium (NBT) test and one patient with congenital neutropenia associated with albinism) but exome sequencing analyses did not yield a molecular diagnosis. We therefore analyzed proteomes of those cases and fitted Gaussian models for each protein using the expression data in 68 healthy samples. Next, we calculated the likelihood that a given expression value in a patient sample could come from the healthy distribution. Finally, we plotted the copy number values for the top ten dysregulated proteins for each case (Fig. 4*A*, 4*B*). The lists of significantly affected proteins and their copy numbers have been reported in the supplemental Table S5.

Fig. 4*A* shows the ten most anomalous protein expression values of neutrophil granulocytes in the CGD patient, with the most pronounced down-regulation of NCF1. Molecular diagnosis of *NCF1* deficiency is challenging, because the human genome contains two *NCF1*-pseudogenes with 99% sequence homology. Using gene- and pseudogene-specific probes (57), two normal NCF1 alleles and four NCF1 pseudogene alleles could be detected in healthy control cells, but only pseudogenes could be detected in patient CGD6 (Fig. 4*C*). This shows that proteome analysis in neutrophil granulocytes could be used as a pre-screening procedure to classify patients for specific *NCF1* sequencing.

Patient SCN7, a 1-year old boy born to consanguineous parents, had immunodeficiency with recurrent infections of skin and the upper respiratory tract, oral ulcerations, splenomegaly and chronic diarrhea, associated with partial albinism. He had intermittent congenital neutropenia and pancytopenia. Proteome analysis of his neutrophil granulocytes revealed a marked decrease of RAB27A protein expression (Fig. 4*B*). Sanger sequencing of the *RAB27a* gene confirmed a segregating homozygous mutation (NM_004580.4(*RAB27A*): c.148_149delinsC) in the patient (Fig. 4*D*) and thus established the diagnosis of Griscelli syndrome (58). Of interest, this patient also showed underexpression of SYTL1, a known binding partner of RAB27A (59).

## DISCUSSION

In this study, we looked at neutrophil proteome changes in patients with monogenic diseases. We show that proteomics is helpful in establishing genetic diagnosis in neutrophil deficiencies in cases where routine genetic testing is not conclusive.

Other laboratories have reported data on the proteome of human neutrophil granulocytes (10–23). Rieckmann *et al.* studied the proteomes of leukocyte subpopulations and reported quantitation data for 6007 proteins in naive neutrophil granulocytes using a data-dependent acquisition method optimized for depth and using TrEMBL protein annotation. Our data-independent approach was aimed at rapid and precise quantification of proteins in a large number of samples and relied on manually curated Swiss-Prot annotation. We observed a moderate level of correlation of our estimated protein copy numbers with the Rieckmann *et al.* data (PCC = 0.7). Moreover, we overcame past problems from endogenous proteases in neutrophils causing extensive sample degradation by enhancing the neutrophil sample handling conditions. This resulted in primarily tryptic peptides which improved reliable protein identification and quantitation. The crucial factor for this improvement seems to be the use of high amounts of protease inhibitors shortly after sample collection.

Our RNAseq data showed high agreement (PCC = 0.85) with publicly available data (56). Interestingly, there was no correlation between protein and mRNA levels in our healthy neutrophil samples (PCC = 0.00), which prompted us to focus our analytical efforts on proteome changes rather than transcriptome changes, as the former is closer related to function (60, 61). This also suggests that clinical neutrophil diagnostics should be rather focused on protein expression analysis than transcriptomics. However, we did not analyze the transcriptomes of patients. There is a possibility that in some cases, such as monogenic diseases affecting the maturation of neutrophils, the correlation between the proteome and the transcriptome is higher and, in those cases, using transcriptomics for clinical diagnostics could be beneficial.

We looked at protein expression profiles of three rare monogenic diseases of neutrophil granulocytes. The largest differences could be observed for the SCN cases. This disease affects the maturation of neutrophils by the mislocalized protease ELANE, leading to increased proteostatic stress within the cell (62). Indeed, we observe an overexpression of the heat-shock response systems which might be an effect of many misfolded proteins within the cell. Increased expression of ribosomal and mitochondrial proteins may be related to the immature phenotype of SCN-affected neutrophils observed in our cytospin controls. We observed decreased expression of proteins in all granule subsets in SCN neutrophils, indicating antimicrobial deficiency beyond reduced cell numbers. This further expands the results of a previous study that analyzed neutrophils of genetically undetermined SCN patients and showed loss of alpha defensins and LL37 (63).

Reduced LL37 (CAMP) level in serum was proposed as a marker for SCN patients, potentially differentiating it from autoimmune neutropenia by indicating faulty granulopoiesis (64). We confirmed the observation of CAMP underexpression in neutrophils of ELANE patients but also showed CAMP underexpression in CGD neutrophils. This warrants caution to the claim that low CAMP levels always indicate disturbance of granulopoiesis.

The proteome changes in the CGD cases were more localized, affecting mostly the protein complex affected by the mutations (flavocytochrome b558) and other proteins localized in secondary granules. In the supplemental Fig. S1 we plotted expression of all neutrophil NADPH oxidase members. Patients with *CYBA* and *CYBB* mutations have low expression of both proteins as has been observed before (65) with no loss of other complex members. It is known that CGD patients suffer from a state of hyperinflammation, but the molecular mechanism has not yet been identified. We did not observe any overexpression of inflammatory mediators and inducers in CGD nor did we see dysregulation of TLRs as mentioned in a previous study (66). One of the LAD cases with *ITGB2* mutation also shows an overexpression of the IFN1 response network. Clinically the patient was very sick which points at a possibility of systemic hyperinflammation. We therefore assumed that the IGN1 response network activation in the analyzed neutrophil proteomes could be related to infection rather than the underlying genetic causes. Analyzing the LAD cases as single samples, we note that proteins with lowest expression levels in LAD1 (*ITGB2* mutation) were the integrins ITGB2, ITGAM and ITGAX whereas in LAD2 (*SLC35C1* mutation) integrin expression was not affected.

Of interest, one of the CGD patients (CGD1) with a splice site mutation showed a protein expression pattern like both CGD and SCN, with milder underexpression of CYBA and CYBB but with substantial underexpression of NCF1 (supplemental Fig. S1). Interestingly, the patient has the same splice site mutation as another CYBB case in our cohort (CGD3) but in addition to immunodeficiency and lack of respiratory burst reaction, shows low absolute neutrophil counts (ANC 500–1400/μl) and a severe neurological phenotype with mental retardation and facial dysmorphia. A polygenic etiology must be suspected, potentially altering his neutrophil proteome in additional ways. An *NCF1* mutation in the patient was excluded by the Gene-scan method (communicated by the treating physician, data not shown). We also excluded Williams Beuren Syndrome, very rarely associated with CGD (67) and found no mutation in XL, thereby excluding McLeod syndrome. The elucidation of causes of the underlying disease warrants a follow-up study.

Finally, data-independent acquisition proteomics helped us guide genetic diagnostics of cases for which standard exome sequencing did not provide clear answers. The observed NCF1 underexpression was a clear hint for the underlying molecular mechanism of the disease as 20% of CGD patient cases are caused by NCF1 mutations. Sequencing efforts for *NCF1* are known to be complicated by two pseudogenes sharing 99% homology. A commercially available solution using a published Gene-scan method (68) can circumvent this problem. However, this is a costly method if applied to all patients. Our proteomics results allowed us to narrow-down the most likely cause of the disease and could be confirmed using the Gene-scan method.

The second case with inconclusive exome sequencing was a patient with neutropenia and partial albinism. Proteomics allowed us to guide genetic analysis and suggested RAB27A protein as the possible cause. Mutations in *RAB27a* are known to cause Griscelli 2 syndrome, matching well with the patient's phenotype. Sanger sequencing of *RAB27a* exons demonstrated a homozygous mutation in exon 2. Indeed, Sanger sequencing and whole exome sequencing confirmed that the patient's mother was heterozygous for this mutation. When we compared the exome result of patient and mother, it became apparent, that in the patient the last part of exon 2 was not covered by the sequencing reads, explaining why we could not detect his mutation initially.

Mass spectrometry is a powerful technique for parallel quantitation of thousands of proteins. Its true potential is only being revealed in the clinical setting. Neutrophils are cells that are hard to work with for molecular diagnostics as they contain numerous highly abundant proteases and RNAses leading to quick degradation of their contents. We presented here a workflow for high purity and high-quality neutrophil proteome samples acquired by state-of-the-art DIA mass spectrometry which allows for quick profiling of neutrophils (1.5 days of sample preparation plus 130 min per sample acquisition time). We believe that this approach can be further used to aid in genetic and clinical diagnosis of neutrophil diseases and expand our knowledge of the neutrophil biology itself.

## DATA AVAILABILITY

The mass spectrometry proteomics data have been deposited to the ProteomeXchange Consortium via the PRIDE partner repository with the data set identifier PXD010701. The raw RNAseq data have been deposited into Gene Expression Omnibus (GEO) under accession number GSE118644.

## Supplementary Material

Supplemental_Table_1

Supplemental_Table_2

Supplemental_Table_3

Supplemental_Table_4

Supplemental_Table_5

Supplemental_Table_7

Supplemental_Table_6

## Footnotes

^1^ The abbreviations used are:

SCN: severe congenital neutropenia
ABC: ammonium bicarbonate
CAN: acetonitrile
ANC: absolute neutrophil counts
CGD: chronic granulomatous disease
DDA: data-dependent acquisition
DIA: data-independent acquisition
DTT: dithiothreitol
EDTA: ethylenediaminetetraacetic acid
FASP: filter-aided sample prep
FDR: false discovery rate
GO: gene ontology
IAA: iodoacetamide
LAD: leukocyte adhesion deficiency
NBT: nitroblue tetrazolium
PBS: phosphate-buffered saline
PCA: principal component analysis
PCC: Pearson correlation coefficient
SCX: strong cation exchange
SDS: sodium dodecyl sulfate
TFA: trifluoroacetic acid.
